# Predictors of mortality in autoimmune disease patients with concurrent cytomegalovirus infections detected by quantitative real-time PCR

**DOI:** 10.1371/journal.pone.0181590

**Published:** 2017-07-25

**Authors:** Kyoung Yong Lee, Byung-Woo Yoo, Sung Soo Ahn, William Han Bae, Hyukmin Lee, Seung Min Jung, Sang-Won Lee, Yong-Beom Park, Jason Jungsik Song

**Affiliations:** 1 Division of Rheumatology, Department of Internal Medicine, Yonsei University College of Medicine, Seoul, South Korea; 2 Department of Laboratory Medicine, Yonsei University College of Medicine, Seoul, South Korea; 3 Institute for Immunology and Immunological Diseases, Yonsei University College of Medicine, Seoul, South Korea; University of San Francisco, UNITED STATES

## Abstract

**Objective:**

Cytomegaloviruses (CMV) can have a significant impact on the prognosis of immunocompromised patients. Unlike in the transplantation and AIDS fields, only a few studies on CMV infections have been published in the field of autoimmunity. In this study, we examined the clinical outcomes of CMV infections in patients with autoimmune diseases at a single tertiary medical institution.

**Methods:**

A retrospective study was performed to identify the mortality risk factors associated with CMV infections in patients with autoimmune diseases. We reviewed the medical records of patients with autoimmune diseases who were diagnosed with CMV infections using real-time quantitative polymerase chain reaction between December 2005 and March 2016. Clinical and laboratory parameters as well as treatment outcomes were analyzed.

**Results:**

Seventy-three CMV infected patients were separated into survivors and non-survivors. Non-survivors had significantly higher median CMV-DNA copy numbers than survivors (95,500 vs 6,700 copies/mL, p = 0.005) and demonstrated significantly more frequent incidents of CMV pneumonitis (69.2 vs 36.2%, p = 0.007). After adjusting for multiple confounding covariates, the log CMV-DNA copies/mL (hazard ratio, 1.48; 95% confidence interval, 1.14–1.92; p = 0.003) and the presence of concurrent infections (hazard ratio, 22.00; 95% confidence interval, 2.75–175.97, p = 0.004) were identified as independent mortality risk factors. Furthermore, patients with high CMV copy numbers (> 60,000 copies/mL) had higher in-hospital mortality than those with low CMV copy numbers (p < 0.05).

**Conclusions:**

CMV-DNA copy numbers and concurrent infections are predictors of in-hospital mortality in CMV-infected patients with autoimmune diseases. Therefore, serial measurements of CMV-DNA copy numbers and close observation for signs of other infections are recommended for patients with autoimmune diseases who have concurrent CMV infection.

## Introduction

Cytomegalovirus (CMV) is a common virus that can adversely affect the prognosis of immunocompromised patients, especially those with AIDS and solid organ or hematopoietic stem cell transplantation [1]. CMV is transmitted via multiple routes, including direct contact with infected saliva or breast milk, sexual transmission, vertical transmission, and blood transfusions. Latent CMV infection may remain dormant throughout the patient’s lifetime [2]. However, when the host immune system deteriorates, the latent virus may reactivate with or without symptoms [3]. CMV is known to damage various organs, including the lung, liver, gastrointestinal tract, bone marrow, and retina.

Although many researchers have investigated the clinical implications of CMV infections in patients that undergo organ transplantation and in those with AIDS [4–6], we still lack an understanding of how CMV infection may affect the outcomes in autoimmune disorders [7]. Patients with autoimmune diseases are particularly susceptible to CMV infection because of their disrupted immune system and frequent immunosuppressive treatments. Furthermore, novel immunosuppressive drugs such as anti-tumor necrosis factor antibodies, Janus kinase inhibitors, and anti-CD20 antibodies increase their risk of infection [8, 9]. Therefore, questions continue to grow regarding the clinical impact of CMV infection in patients with autoimmune diseases.

CMV quantitative real-time polymerase chain reaction (PCR) is the preferred method for the diagnosis of CMV infection [10]. It is a very sensitive assay capable of detecting and quantifying even a small amount of viral nucleic acids [11]. However, when CMV PCR tests are positive, the technique is unable to determine whether the infection is latent or has developed into an active end-organ disease. Furthermore, we still lack clear guidelines in the management of CMV infections in patients with autoimmune diseases. In this retrospective observational study, we evaluated the association between CMV infections and survival outcomes in hospitalized patients with autoimmune diseases and identified the risk factors for in-hospital mortality in this population.

## Materials and methods

### Study design and patient selection

This is a retrospective observational study over a 10-year period at a single tertiary medical institution to identify the mortality risk factors associated with CMV infections in patients with autoimmune diseases. We reviewed the medical records of hospitalized patients with autoimmune diseases who were evaluated for CMV infections because of clinical suspicion at the Yonsei University Severance Hospital (Seoul, South Korea) between December 1, 2005, and March 31, 2016. Patients aged 18–85 were included in our study. Patients were excluded if they had also been diagnosed with cancer or HIV infection and/or if they had undergone organ or hematopoietic stem cell transplantation. This study was approved by the Institutional Review Board at Severance Hospital (IRB approval number 4-2016-1154) and was conducted in accordance with the principles set forth in the Declaration of Helsinki. The requirement to obtain informed consent was waived because of the retrospective nature of the study.

### Data collection

We collected the following data for each patient: age, sex, disease duration, white blood cell and differential counts, hemoglobin level, platelet count, total protein and albumin levels, erythrocyte sedimentation rate, C-reactive protein level, CMV-DNA copy numbers, clinical manifestations, comorbid conditions, medications, clinical outcomes, ventilator use, and presence of other infections. When CMV-DNA copy numbers were measured more than once, the highest copy numbers were used for analysis. Laboratory data collected on the day with the highest recorded CMV-DNA copy numbers were used. The mean daily steroid dose was calculated using the accumulated steroid doses within 30 days from the day the highest CMV-DNA copy number was reported. All steroid doses were converted to equivalent doses of methylprednisolone.

### Definitions

CMV infection was defined as the detection of CMV PCR positivity in the blood or body fluids. CMV disease was defined as a condition in which the organ involvement of CMV infection was confirmed. Asymptomatic CMV infection was defined as a condition in which the patient showed CMV PCR positivity with no symptoms or organ involvement.

### Quantitative PCR for CMV

To perform quantitative real-time PCR for CMV, DNA was extracted from whole blood using a QIAamp DSP DNA Mini Kit (Qiagen, Germany) and QIAcube (Qiagen, Germany) in accordance with the manufacturer’s instructions. Real-time PCR for CMV-DNA was performed using the LightCycler 480 (Roche Diagnostics, Germany) and Bio-Core CMV Quantification real-time PCR kit (Bio-Core, Korea). For the standardization of the results, the World Health Organization International Standard for human CMV for nucleic acid amplification techniques was used. The data were reported as copies/mL.

### Statistical analysis

The Statistical Analysis System (SAS) version 9.4 software (SAS, Cary, NC, USA) was used for all data analyses. Non-normally distributed data are presented as medians (interquartile ranges) and categorical variables are expressed as counts (%). For continuous variables, group comparisons were performed using the Mann-Whitney *U* test. Categorical data were compared using the chi-squared or Fisher’s exact tests. Risk factors for in-hospital mortality in patients with autoimmune diseases and concurrent CMV infections were identified using univariate and multivariate logistic regression analyses, with the hazard ratios (HRs) listed along with 95% confidence intervals (CIs). For multivariate analysis, factors that were statistically significant in the univariate logistic model were used. The maximal cut-off value for the CMV-DNA copy number to predict in-hospital mortality was calculated using area under the receiver operating characteristic (ROC) curve analyses and the survival rates of patients were compared using the Kaplan-Meier method. In all statistical analyses, a two-tailed p-value <0.05 was considered statistically significant.

## Results

### Patient baseline characteristics

We identified 73 CMV PCR positive and 123 CMV PCR negative autoimmune patients. Mortality rate was higher in the CMV PCR positive patients than in the CMV PCR negative patients (35.6% vs 11.4%, p < 0.001, S1 Table). Among the 73 CMV PCR positive patients enrolled in the further analysis, the median age was 58 years; 25 were male and 48 were female; the median disease duration was 3 years (range, 1–10). Autoimmune diseases consisted of 28 cases (38.4%) of systemic lupus erythematosus (SLE), 18 cases (24.6%) of rheumatoid arthritis (RA), 12 cases (16.4%) of vasculitis, 9 cases (12.3%) of inflammatory myositis, 3 cases (4.1%) of Behçet disease, and 3 cases (4.1%) of adult-onset Still disease. Non-survivors had significantly higher mean CMV-DNA copy numbers than survivors (p = 0.005). In addition, non-survivors had significantly lower total protein (p = 0.025) and albumin (p = 0.007) levels than survivors (Table 1).

**Table 1:** Baseline characteristics according to disease outcome in CMV PCR positive patients.

	Non-survivors (n = 26)	Survivors (n = 47)	p-value
Age (years)	64.0 (49.0–72.0)	58.0 (43.2–66.0)	0.271
Female sex, n (%)	17 (65.3)	31 (65.9)	0.960
CMV-DNA (copies/mL)	95,500.0 (5150.0–315,000.0)	6700.0 (3995.0–40,950.0)	0.005
WBC count (/μL)	8620.0 (5490.0–12,480.0)	8270.0 (4940.0–14,020.0)	0.670
Neutrophil count (/μL)	8045.0 (4860.0–11,210.0)	7210.0 (4090.0–12,330.0)	0.397
Lymphocyte count (/μL)	415.0 (260.0–620.0)	420.0 (190.0–890.0)	0.895
Hemoglobin (g/dL)	10.0 (9.0–10.0)	10.0 (8.0–11.0)	0.683
Platelet count (/10^3^ μL)	129.0 (77.0–242.0)	198.0 (119.0–322.0)	0.106
BUN (mg/dL)	29.0 (18.0–47.0)	21.0 (11.0–34.0)	0.040
Cr (mg/dL)	1.0 (1.0–2.0)	1.0 (1.0–1.0)	0.444
Total protein (mg/dL)	5.0 (4.0–5.0)	5.0 (5.0–6.0)	0.025
Albumin (mg/dL)	2.0 (2.0–3.0)	3.0 (2.0–3.0)	0.007
ESR (mm/hr)	27.0 (6.0–92.0)	53.0 (17.0–83.0)	0.284
CRP (mg/L)	54.0 (31.0–97.0)	52.0 (5.0–114.0)	0.565

Values are expressed as medians (Q1–Q3) or counts (%). P-values are based on the Mann-Whitney *U* test, chi-squared test, or Fisher’s exact test. BUN, blood urea nitrogen; CMV, cytomegalovirus; Cr, creatinine; CRP, C-reactive protein; ESR, erythrocyte sedimentation rate; WBC, white blood cell.

### Clinical features of CMV infections

CMV end-organ diseases consisted of pneumonitis, retinitis, enteritis, and hepatitis; 73.1% of non-survivors and 44.8% of survivors had CMV end-organ diseases while 26.9% of non-survivors and 55.3% of survivors had asymptomatic CMV infections. Survivors had fewer cases of CMV pneumonitis (p = 0.007) and more cases of asymptomatic CMV infection (p = 0.020) than non-survivors. Non-survivors required mechanical ventilation more frequently than did survivors (p < 0.001). The frequency of ganciclovir treatment was comparable in the two groups (Table 2). We further evaluated whether a higher mortality rate is associated with certain autoimmune diseases. The majority of patients were diagnosed as having SLE, RA, vasculitis, and myositis (Table 2). There was no difference in mortality rate in specific autoimmune diseases.

**Table 2:** Clinical features of cytomegalovirus (CMV) infections.

	Non-survivors (n = 26)	Survivors (n = 47)	p-value
**Organ involvement**			
CMV pneumonitis	18 (69.2)	17 (36.2)	0.007
CMV colitis	0 (0.0)	2 (4.3)	0.535
CMV retinitis	0 (0.0)	2 (4.3)	0.535
CMV hepatitis	1 (3.9)	0 (0.0)	0.356
Asymptomatic CMV infection	7 (26.9)	26 (55.3)	0.020
**Treatment**			
Use of ganciclovir	15 (57.7)	20 (42.6)	0.215
Use of ventilator care	21 (80.8)	2 (4.3)	<0.001
**Type of autoimmune diseases**			0.435
Systemic lupus erythematosus	7 (26.9)	21 (44.7)	
Rheumatoid arthritis	7 (26.9)	11 (23.4)	
Vasculitis	5 (19.2)	7 (14.9)	
Inflammatory myositis	5 (19.2)	4 (8.5)	
Adult-onset Still disease	2 (7.7)	1 (2.1)	
Behçet disease	0 (0.0)	3 (6.4)	

Values are expressed as counts (%). P-values were calculated with the chi-squared or Fisher’s exact tests.

### Immunosuppressive treatments

There were no significant differences in the types of drugs used by the survivors and non-survivors (Table 3). The median steroid dose was not significantly different in the two groups (32.07 vs 24 mg/day). The proportion of patients taking cyclophosphamide or other immunosuppressants was also comparable in the two groups.

**Table 3:** Medications administered to patients in the survivor and non-survivor groups.

	Non-survivors (n = 26)	Survivors (n = 47)	p-value
**Steroids**			
Steroid dose (mg/day)	32.1 (17.3–49.5)	24.0 (8.0–75.9)	0.256
Steroid only	5 (19.2)	16 (34.0)	0.181
Steroid plus cyclophosphamide	4 (15.4)	5 (10.6)	0.712
Steroid plus other immunosuppressants	17 (65.4)	26 (55.3)	0.403
**Immunosuppressants**			
Cyclophosphamide	4 (15.4)	5 (10.6)	0.712
Methotrexate	4 (15.4)	9 (19.2)	0.760
Leflunomide	1 (3.9)	0 (0.0)	0.356
Azathioprine	3 (11.5)	9 (19.2)	0.519
Mycophenolate mofetil	3 (11.5)	4 (8.5)	0.694
Calcineurin inhibitors	1 (3.9)	2 (4.3)	0.999
Rituximab	2 (7.7)	1 (2.1)	0.287
Tumor necrosis factor inhibitors	1 (3.9)	0 (0.0)	0.356
None	2 (7.7)	1 (2.1)	0.287

Values are expressed as medians (Q1–Q3) or counts (%). P-values are based on the Mann-Whitney *U* test, chi-squared test, or Fisher’s exact test. Values are expressed as counts (%). P-values were calculated with the chi-squared or Fisher’s exact tests.

### Evaluation of concurrent infections

There was no significant difference in the number of patients infected by each bacterial strain identified in the two groups (Table 4). However, non-survivors were infected more frequently by multiple microbes than survivors (57.7% vs 27.7%, p = 0.012). *Pneumocystis jiroveci* infection was twice as common in non-survivors as in survivors, although the difference was not statistically significant.

**Table 4:** Comparison of microbes in the survivor and non-survivor groups.

	Non-survivors (n = 26)	Survivors (n = 47)	p-value
**Type of infection**			
Single organism	10 (38.5)	12 (25.5)	0.249
Multiple organisms	15 (57.7)	13 (27.7)	0.012
No organism	1 (3.8)	22 (46.8)	<0.001
**Identified pathogens**			
*Acinetobacter baumannii*	7 (26.9)	5 (10.6)	0.101
*Enterococcus* species	5 (19.2)	3 (6.4)	0.124
*Escherichia coli*	0 (0)	3 (6.4)	0.548
*Klebsiella pneumoniae*	0 (0)	3 (6.4)	0.548
*Pseudomonas aeruginosa*	3 (11.5)	2 (4.3)	0.340
*Staphylococcus aureus*	1 (3.8)	1 (2.1)	1.000
*Clostridium difficile*	1 (3.8)	3 (6.4)	1.000
*Pneumocystis jiroveci*	9 (34.6)	8 (17.0)	0.089
*Aspergillus* species	6 (23.1)	9 (19.1)	0.696

Values are expressed as counts (%). P-values were calculated with the chi-squared or Fisher’s exact tests.

### Mortality risk factors

We utilized univariate analyses to determine whether any clinical features of the patients increased the mortality risk. To calculate the validity and significance of the risk factors, we performed a logistic regression analysis. The log CMV-DNA copy number (univariate HR, 1.479; 95% CI, 1.140–1.918; p = 0.003, multiple 1 HR, 1.415; 95% CI, 1.027–1.950; p = 0.034, multiple 2 HR, 1.409; 95% CI, 1.016–1.954; p = 0.040) and the presence of concurrent infections (univariate HR, 22.000; 95% CI, 2.750–175.971; p = 0.004, multiple 1 HR, 16231; 95% CI, 1.846–142.681; p = 0.012, multiple 2 HR, 18.425; 95% CI, 2.109–161.006; p = 0.008) were identified as significant mortality risk factors (Table 5).

**Table 5:** Survival outcomes associated with mortality risk factor among cytomegalovirus (CMV)-infected patients.

	Univariate HR (95% CI)	p-value	Multivariate 1 HR (95% CI)	p-value	Multivariate 2 HR (95% CI)	p-value
Age	1.012 (0.981–1.044)	0.441	0.998 (0.957–1.041)	0.924	1.004 (0.964–1.045)	0.844
Female sex	1.026 (0.374–2.812)	0.961	1.082 (0.284–4.123)	0.908	1.037 (0.274–3.921)	0.958
Other infections	22.000 (2.750–175.971)	0.004	16.231 (1.846–142.681)	0.012	18.425 (2.109–161.006)	0.008
Log CMV PCR	1.479 (1.140–1.918)	0.003	1.415 (1.027–1.950)	0.034	1.409 (1.016–1.954)	0.040
Ganciclovir	0.543 (0.206–1.432)	0.217	0.864 (0.226–3.309)	0.864	0.815 (0.221–3.008)	0.759
Pulsed MPSL	0.948 (0.337–2.667)	0.920	1.331 (0.259–6.847)	0.732	1.548 (0.287–8.334)	0.611
Oral steroid	1.000 (0.999–1.001)	0.813	0.993 (0.982–1.004)	0.199	0.994 (0.983–1.005)	0.271
Log WBC count	1.384 (0.706–2.713)	0.345	1.342 (0.620–2.904)	0.455		
Log Lymphocyte	1.153 (0.819–1.623)	0.414			1.319 (0.673–2.588)	0.420

Values are expressed as HR (95% CI). P-values were calculated using logistic regression models. CI, confidence interval; HR, hazard ratio; MPSL, methylprednisolone; WBC, white blood cell.

**Fig 1:**
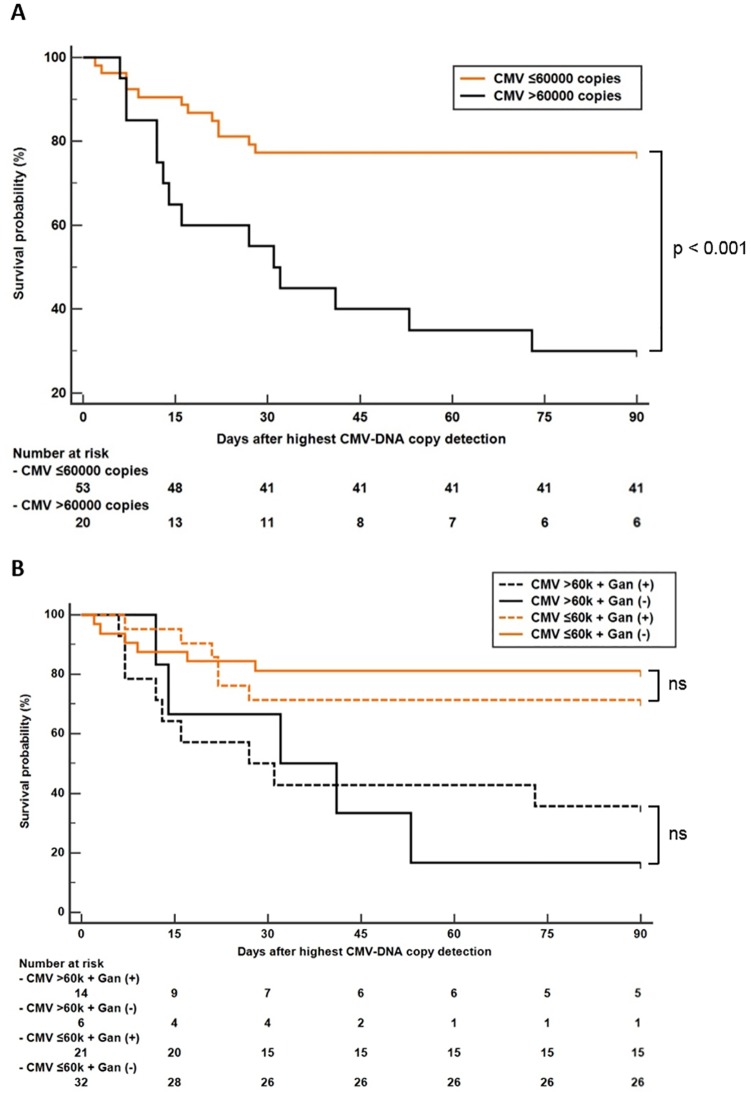
Kaplan-Meier survival analysis of patients with autoimmune disease and CMV infection according to CMV-DNA copy number. (a) Survival analysis according to CMV titer. (b) Subgroup survival analysis according to ganciclovir (Gan) treatment. Cut-off value 60,000 copies/mL, log-rank test p < 0.001.

Area under the ROC curve analysis revealed that CMV-DNA copy number > 60,000 copies/mL was the best cut-off for predicting in-hospital mortality in patients with CMV infection. Kaplan-Meier analysis with the log-rank test showed that the in-hospital mortality rate was higher in patients with CMV-DNA copy numbers greater than 60,000 copies/mL (p < 0.05) (Fig 1a). We further analyzed patients’ survival according to ganciclovir treatment. Because CMV PCR test was performed multiple times in the same patient, the highest copy number was used for the survival analysis. The highest CMV-DNA copy number was observed before ganciclovir treatment in all patients, except in one patient, who had highest copy number after ganciclovir treatment. Although the survival probability of patients with ganciclovir treatment was higher than that of patients without ganciclovir treatment in the high-titer CMV-DNA patient group, the survival benefit of ganciclovir was insignificant in both the high- and the low-titer CMV groups (Fig 1b).

### Effect of ganciclovir treatment on the CMV-DNA copy number

Thirty-five (48%) patients received ganciclovir, which included 8 patients who had missing CMV-DNA copy number follow-up data. In the remaining 27 patients, ganciclovir treatment reduced the CMV-DNA copy number in both the survivor and non-survivor groups (Table 6). However, the negative conversion rate was lower in the non-survivor group than in the survivor group (p = 0.023).

**Table 6:** Effect of ganciclovir treatment on the CMV-DNA copy number.

	Survivor (n = 15)	Non-survivor (n = 12)	p-value
CMV-DNA copy number before ganciclovir treatment	39,750.0 (13,300.0–88,875.0)	186,500.0 (8375.0–551,500.0)	0.525
CMV-DNA copy number after ganciclovir treatment	0.0 (0.0–0.0)*	832.5 (0.0–5652.5)**	0.013
Ganciclovir treatment duration (days)	20.0 (10.3–21.5)	13.5 (12.5–16.0)	0.180
Negative conversion rate (%)	14 (93.3)	6 (50.0)	0.023

*p = 0.001, CMV-DNA copy number before and after ganciclovir treatment in survivors.

**p = 0.005, CMV-DNA copy number before and after ganciclovir treatment in non-survivors.

## Discussion

Latent CMV infection may persist without causing any harm in healthy individuals [1]. Even though it has been widely accepted that CMV infection may lead to severe or even fatal illnesses in immunocompromised conditions, such as HIV infection, hematologic malignancies, organ transplantation, or immunosuppressive therapy [3], only a few studies have investigated the association between CMV infection and patient survival in autoimmune disorders [12–14]. In our study involving a 10-year period in a single tertiary medical institute, we demonstrated that the mortality rate was significantly higher in patients with autoimmune diseases diagnosed with CMV infections. Furthermore, the incidence of CMV pneumonitis was significantly higher in non-survivors than in survivors. Our data do not prove that CMV infection is the direct cause of mortality because 26.9% of non-survivors had asymptomatic CMV infection. Even for patients with CMV end-organ diseases, it is difficult to identify the primary cause of death in cases of CMV infection in the presence of autoimmune diseases because the patients often have multiple medical conditions, including systemic inflammation due to autoimmune diseases, and other concurrent infections. However, it is important to understand the risk factors for mortality when patients with autoimmune diseases are infected with CMV.

CMV PCR is a widely available rapid and sensitive technique for CMV detection [15]. CMV-DNA copy number is a useful marker to predict the progression of CMV infection [16]. It has been used to determine the response to treatment and to identify patients at risk of CMV disease after transplantation for preemptive therapy [15, 17]. In the present study, we found that the CMV-DNA copy numbers were significantly higher in non-survivors than in survivors (Table 1). Consistent with this finding, the Kaplan-Meier curves demonstrated a lower survival rate in patients with CMV-DNA copy numbers greater than 60,000 copies/mL (Fig 1). Univariate and multivariate logistic regression analyses for in-hospital mortality revealed that log CMV-DNA copy number was a significant independent mortality risk factor. Interestingly, however, Takizawa et al. [14] reported that CMV pp65 antigenemia did not cause significant increases in mortality risk among 151 Japanese patients with autoimmune diseases among whom 50% had SLE. Rather, other factors such as lymphopenia, infectious complications, and clinical symptoms were significant risk factors. Similar to our findings, Tsai et al. [18] reported that CMV pp65 antigenemia or lymphopenia predicted mortality or morbidity in 54 Chinese patients with autoimmune diseases, among whom 70% had SLE. Because CMV pp65 antigenemia detection is labor-intensive and is limited by the need for immediate processing and subjective bias in interpretation, most laboratories are now using quantitative real-time PCR based technologies [19]. Our study differs from previous studies, given that we evaluated the association between in-hospital mortality and high CMV-DNA copy numbers, which is more relevant to current practice pattern. Another study by Dodt et al. [4] demonstrated that lymphopenia and leukopenia significantly increased the mortality rate of patients with AIDS. In our study, however, neither lymphopenia nor leukopenia appeared to be a significant risk factor. This discrepancy might be due to differences in the underlying diseases. In our study, total protein and albumin levels were lower in the non-survivors than in the survivors. Interestingly, Borthakur et al. [20] also reported that low serum albumin was associated with CMV reactivation in patients with leukemia who were treated with anti-CD52 antibody. Low serum albumin could be associated with susceptibility to CMV infection due to low nutritional or general immune status.

Current CMV anti-viral treatment recommendations are based on the clinical manifestations of the infection. Since potent immunosuppressive treatment can trigger CMV reactivation, it is clinically challenging to distinguish CMV infection from CMV disease in patients with autoimmune diseases. To make the situation more complex, there can be additional opportunistic infections or hospital-acquired bacterial infections in febrile patients with autoimmune diseases who receive long-term immunosuppressive therapy. Pre-emptive anti-viral treatment has proven effective in lowering the incidence and mortality rates of CMV disease in patients that undergo transplantation [5, 6, 10]. Ganciclovir treatment did not significantly improve survival outcomes in patients with autoimmune diseases in our study (Table 5), but it was effective in reducing CMV-DNA titer in both the survivor and non-survivor groups (Table 6). Therefore, CMV may not be a major factor associated with mortality of autoimmune patients. It is possible that the elevation of CMV-DNA copy numbers occurred secondary to severe immune suppression in our patients. As patients with autoimmune diseases have dysregulated immune systems, which often require frequent immunosuppressive treatments, they are susceptible to infection. Given that 96.2% of non-survivors had other bacterial or fungal infections while only 53.2% of survivors had other infections (Table 4), concurrent infections appeared to have contributed to mortality in our patients. Univariate analysis consistently indicated that concurrent bacterial or fungal infections had a HR of 22 (Table 5; p = 0.004), which is greater than the HR for CMV-DNA copy number (HR = 1.479, p = 0.003).

There are factors that we could not control in our study in demonstrating the treatment benefit on survival such as (i) small sample size for subgroup analysis, (ii) suboptimal CMV treatment for the non-survival group, and (iii) heterogeneity of concurrent infection. In fact, subgroup analysis on high-titer CMV-DNA patients demonstrated that the survival probability of patients with ganciclovir treatment was higher than that of patients without ganciclovir treatment (Fig 1b), but it was not statistically significant, which is possibly due to small sample size. The CMV negative conversion rate after ganciclovir treatment was significantly lower in the non-survivor group (Table 6), which suggests that the CMV treatment was not optimal in the non-survivor group. Given that only 57.7% of the non-survivors had received ganciclovir (Table 2), a delay in the detection of CMV infection in non-survivors is also possible. Additionally, because treatments of other concurrent infections are also important for survival of our patients with autoimmune diseases, it is difficult to evaluate the survival benefits of ganciclovir with heterogeneity of concurrent infections. Prospective studies with larger populations are necessary to evaluate whether anti-viral treatment reduces mortality in high-risk patients.

There are several limitations to our study. First, as it is a retrospective cohort study, there could be selective bias in collecting relevant information from the electronic medical records. Second, our study population might have been skewed toward those with poor outcomes because CMV PCR tests were usually performed when the clinical course of the patient worsened. These factors might have influenced the high mortality rate observed in CMV-DNA—positive patients. Third, treatments for CMV disease were not controlled. Fourth, we were not able to determine if the CMV infection detected in our patients was due to truly latent virus or due to replicating but asymptomatic virus because we did not evaluate the CMV gene expression data such as the expression of CMV latent genes in the absence of CMV lytic genes. Lastly, the stage of CMV infection (primary vs recurrent) was not evaluated.

In summary, the present study revealed a significant association between CMV-DNA copy numbers, other bacterial and fungal infections, and mortality rates in autoimmune patients with concurrent CMV infection. Therefore, we recommend evaluations for CMV end-organ diseases such as CMV pneumonitis, serial follow-ups to monitor CMV-DNA copy numbers, and surveillance for other bacterial and fungal infections in CMV PCR positive patients with autoimmune diseases.

## Supporting information

S1 File The datasets for Kaplan-Meier analysis. (XLSX)

S2 File The datasets for baseline characteristics comparison. (XLSX)

S1 Table Demographics of the CMV PCR positive and CMV PCR negative groups. (DOCX)
