# Supplementary material for: Predictors of mortality in autoimmune disease patients with concurrent cytomegalovirus infections detected by quantitative real-time PCR
Source: PLoS One. 2017 Jul 25;12(7):e0181590. doi: 10.1371/journal.pone.0181590 (PMC5526501; doi:10.1371/journal.pone.0181590)
Supplement: S1 Table — (DOCX) [file pone.0181590.s003.docx]

**S1 Table. Demographics of the CMV PCR positive and CMV PCR negative groups.**

|  | **CMV positive**  **(n=73)** | **CMV negative (n=123)** | **p-value** |
| --- | --- | --- | --- |
| Female, n (%) | 48 (65.8) | 92 (74.8) | 0.177 |
| Age, years | 58.0 (43.7-69.0) | 53.0 (38.2-65.0) | 0.068 |
| Systemic lupus erythematosus, n (%) | 28 (38.4) | 34 (27.6) | 0.120 |
| Rheumatoid arthritis, n (%) | 18 (24.7) | 38 (30.9) | 0.351 |
| Others, n (%) | 27 (37.0) | 51 (41.5) | 0.537 |
| 90-Day mortality, n (%) | 26 (35.6) | 14 (11.4) | <0.001 |

Values are expressed as medians (Q1–Q3) or counts (%). P-values are based on the Mann-Whitney U test or chi-squared test. CMV, cytomegalovirus.
